# Supplementary material for: The Impact and Progression of the COVID-19 Pandemic in Bulgaria in Its First Two Years
Source: Vaccines (Basel). 2022 Nov 10;10(11):1901. doi: 10.3390/vaccines10111901 (PMC9696094; doi:10.3390/vaccines10111901)
Supplement: Supplementary file 1 [file vaccines-10-01901-s001.zip › vaccines-2000899-supplementary.pdf]

Supplementary Figures

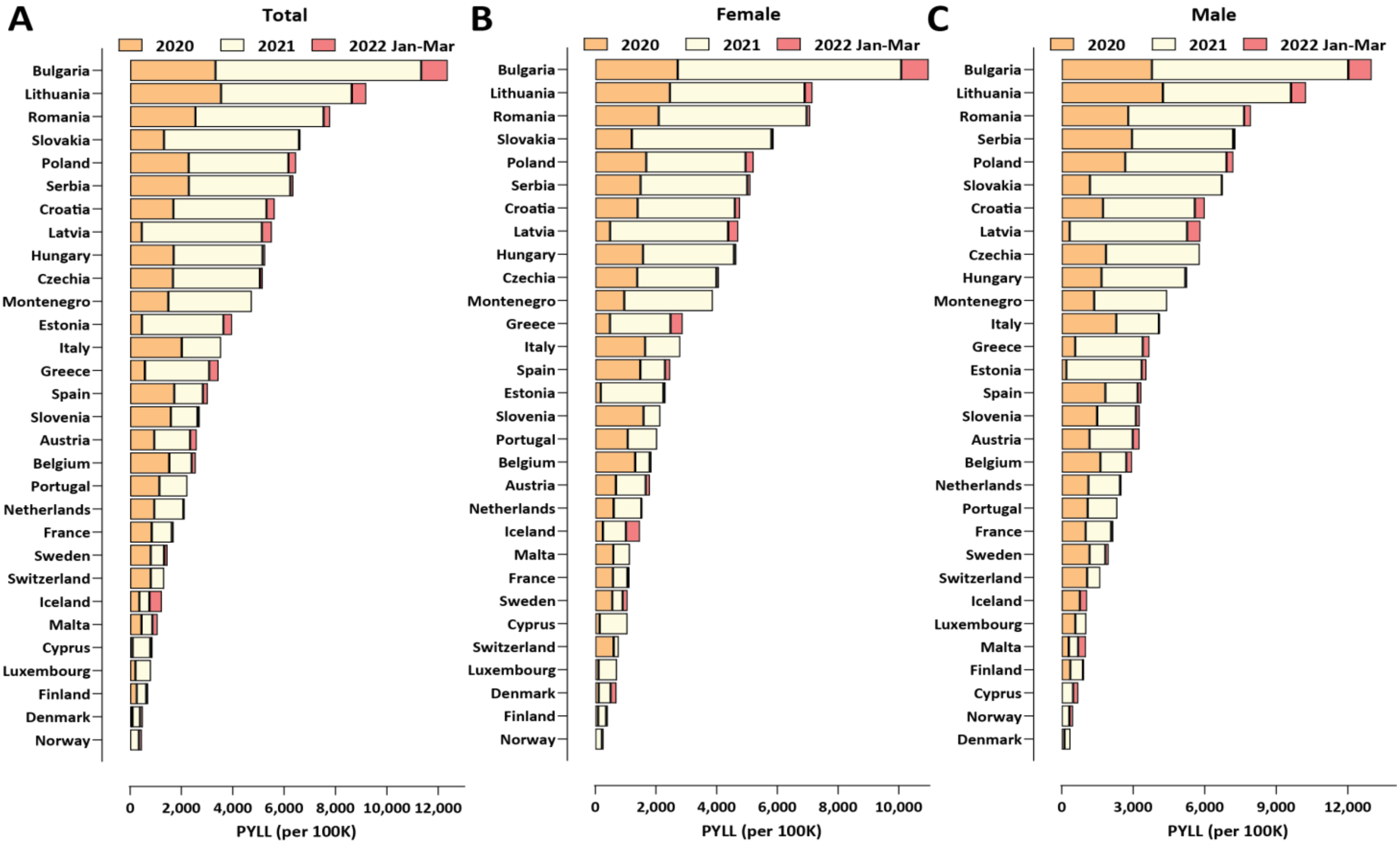

Supplementary Figure S1: Excess mortality in Europe and Bulgaria during the COVID-19 pandemic (up to the end of March 2022). (A) Standardized PYLL values, total; (B) Standardized PYLL values, females; (C) Standardized PYLL values, males.

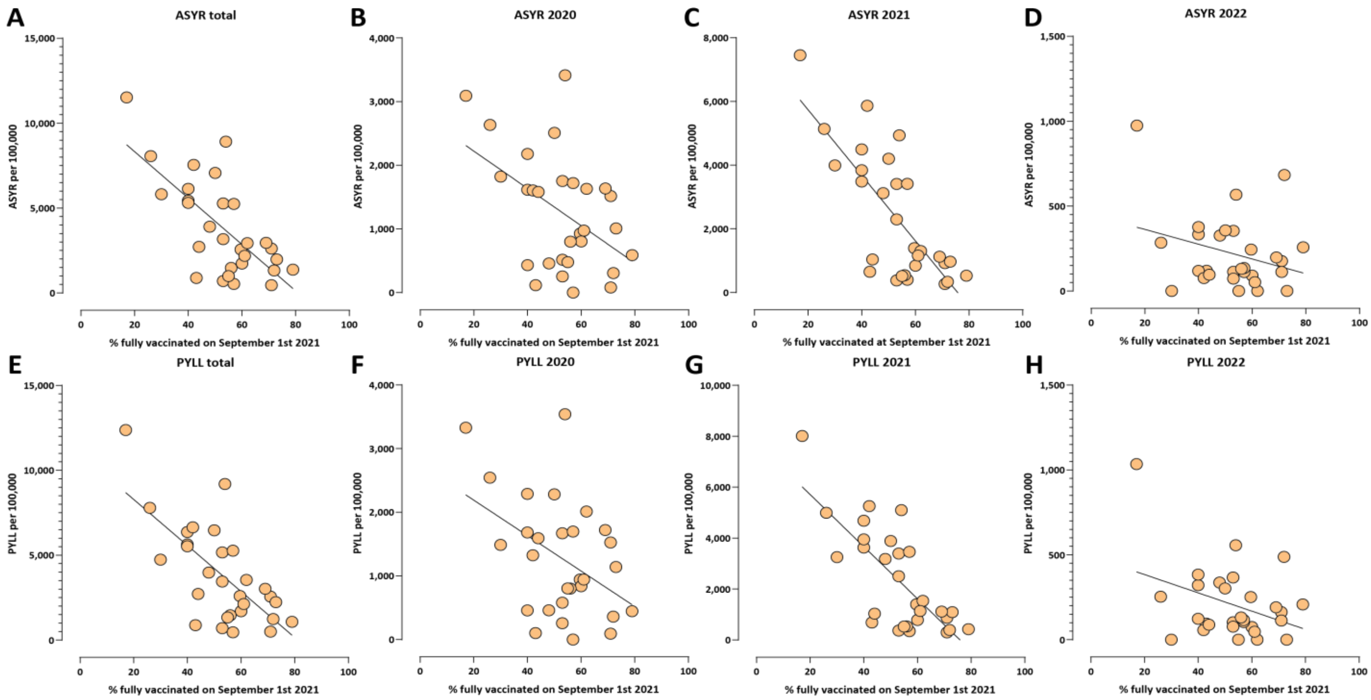

Supplementary Figure S2: Correlation between rates of full vaccination and ASYR and PYLL excess mortality measures in European countries.

(A) ASYR, total (Pearson $R^2 = 0.5$, $p \leq 0.0001$; Spearman $r = -0.64$, $p = 0.0002$);

(B) ASYR, 2020 (Pearson $R^2 = 0.22$, $p = 0.0105$; Spearman $r = -0.36$, $p = 0.0549$);

(C) ASYR, 2021 (Pearson $R^2 = 0.57$, $p \leq 0.0001$; Spearman $r = -0.69$, $p \leq 0.0001$);

(D) ASYR, 2022 (Pearson $R^2 = 0.08$, $p = 0.1286$; Spearman $r = -0.2$, $p = 0.2982$);

(E) PYLL, total (Pearson $R^2 = 0.47$, $p \leq 0.0001$; Spearman $r = -0.62$, $p = 0.0003$);

(F) PYLL, 2020 (Pearson $R^2 = 0.19$, $p = 0.0169$; Spearman $r = -0.32$, $p = 0.08$);

(G) PYLL, 2021 (Pearson $R^2 = 0.56$, $p \leq 0.0001$; Spearman $r = -0.65$, $p = 0.0001$);

(H) PYLL, 2022 (Pearson $R^2 = 0.13$, $p = 0.0523$; Spearman $r = -0.19$, $p = 0.3106$).

	ASYR			PYLL		
	all	male	female	all	male	female
Austria	12.17	14.38	8.98	12.32	13.38	10.36
Belgium	11.84	14.05	8.31	11.59	12.49	9.23
Bulgaria	11.70	12.70	10.43	12.57	12.02	12.51
Croatia	11.33	12.99	9.53	11.75	11.51	11.23
Cyprus	16.54	n/a	24.30	16.48	n/a	22.38
Czechia	12.84	14.48	10.70	12.58	12.57	11.67
Denmark	10.29	7.62	16.74	11.26	8.04	17.70
Estonia	14.32	17.65	9.17	14.59	15.29	11.52
Finland	15.56	19.03	7.86	15.56	17.64	10.08
France	12.71	14.37	9.31	12.47	12.82	10.31
Greece	12.94	13.84	10.61	14.10	14.07	12.60
Hungary	14.97	15.98	13.21	15.02	14.18	14.57
Iceland	24.40	27.33	22.86	22.87	23.46	21.12
Italy	9.47	11.27	7.52	11.45	12.21	10.37
Latvia	13.20	17.17	9.07	13.74	15.42	11.50
Lithuania	13.94	16.27	10.41	14.38	14.57	12.40
Luxembourg	27.49	26.56	18.90	27.01	25.21	19.48
Malta	12.83	18.13	10.36	10.07	13.11	9.41
Montenegro	13.37	13.95	12.22	10.90	9.74	10.96
Netherlands	12.00	12.96	11.03	11.68	11.56	11.56
Poland	14.80	16.99	12.03	13.54	13.47	12.39
Portugal	9.31	11.19	7.96	10.51	11.03	10.26
Romania	13.10	14.08	12.00	12.66	11.86	12.93
Serbia	12.30	13.17	11.05	12.75	12.48	12.46
Slovakia	15.55	17.54	13.01	13.68	13.41	12.82
Slovenia	11.34	14.53	7.70	11.34	12.12	9.29
Spain	12.62	13.93	10.19	12.91	12.83	11.63
Sweden	11.27	13.38	8.34	11.19	12.34	9.38
Switzerland	7.53	9.76	3.50	10.07	11.23	7.18

Supplementary Figure S3: Year of life lost per excess death in European countries. Note that the very high values for a few countries (e.g. Cyprus, Iceland, Luxembourg, Finland, Malta) might be artifacts resulting from significantly insignificant excess deaths (z-score significantly below 2).

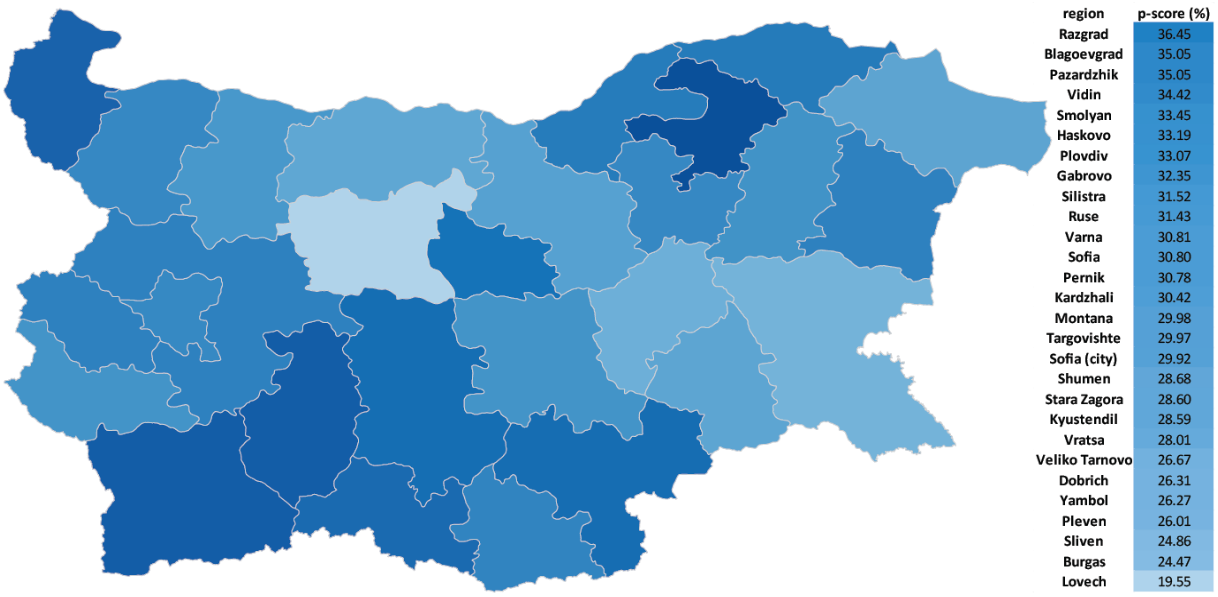

Supplementary Figure S4: Excess mortality in Bulgarian regions (P-scores).

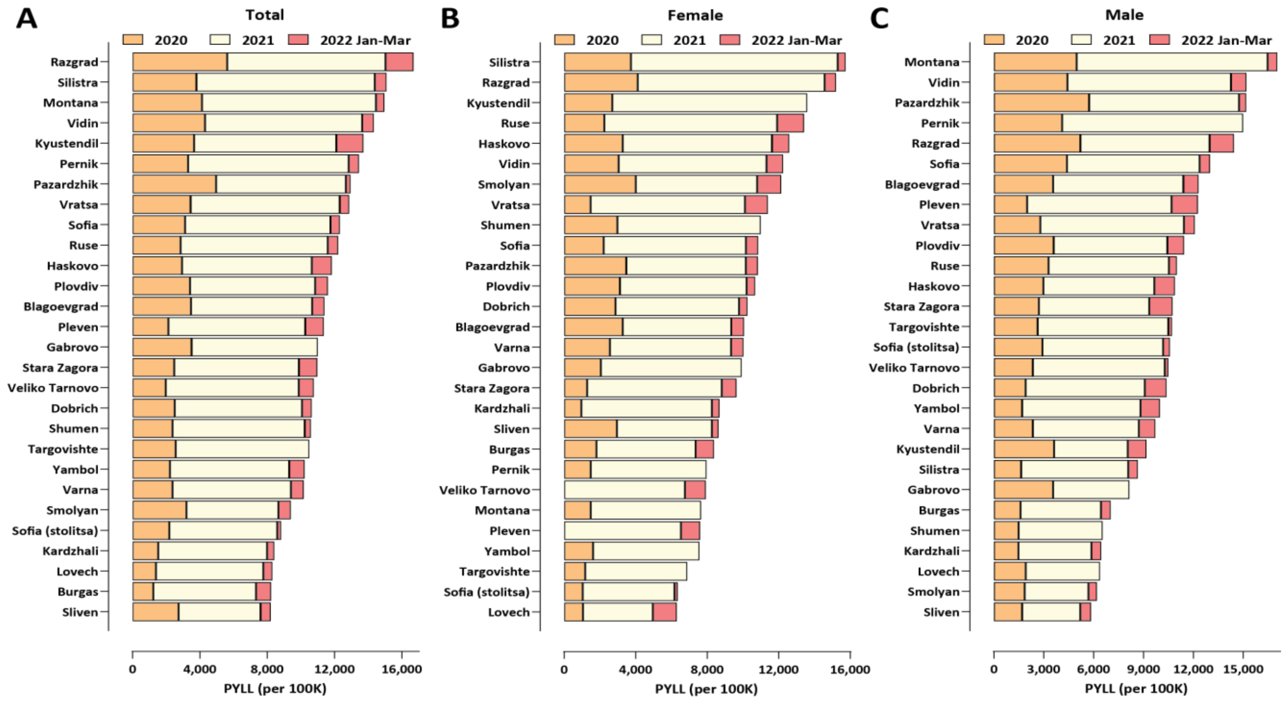

Supplementary Figure S5: Regional excess mortality patterns in Bulgaria during the COVID-19 pandemic (up to the end of March 2022). (A) Total, standardized PYLL values; (B) Female, standardized PYLL values; (C) Male, standardized PYLL values.

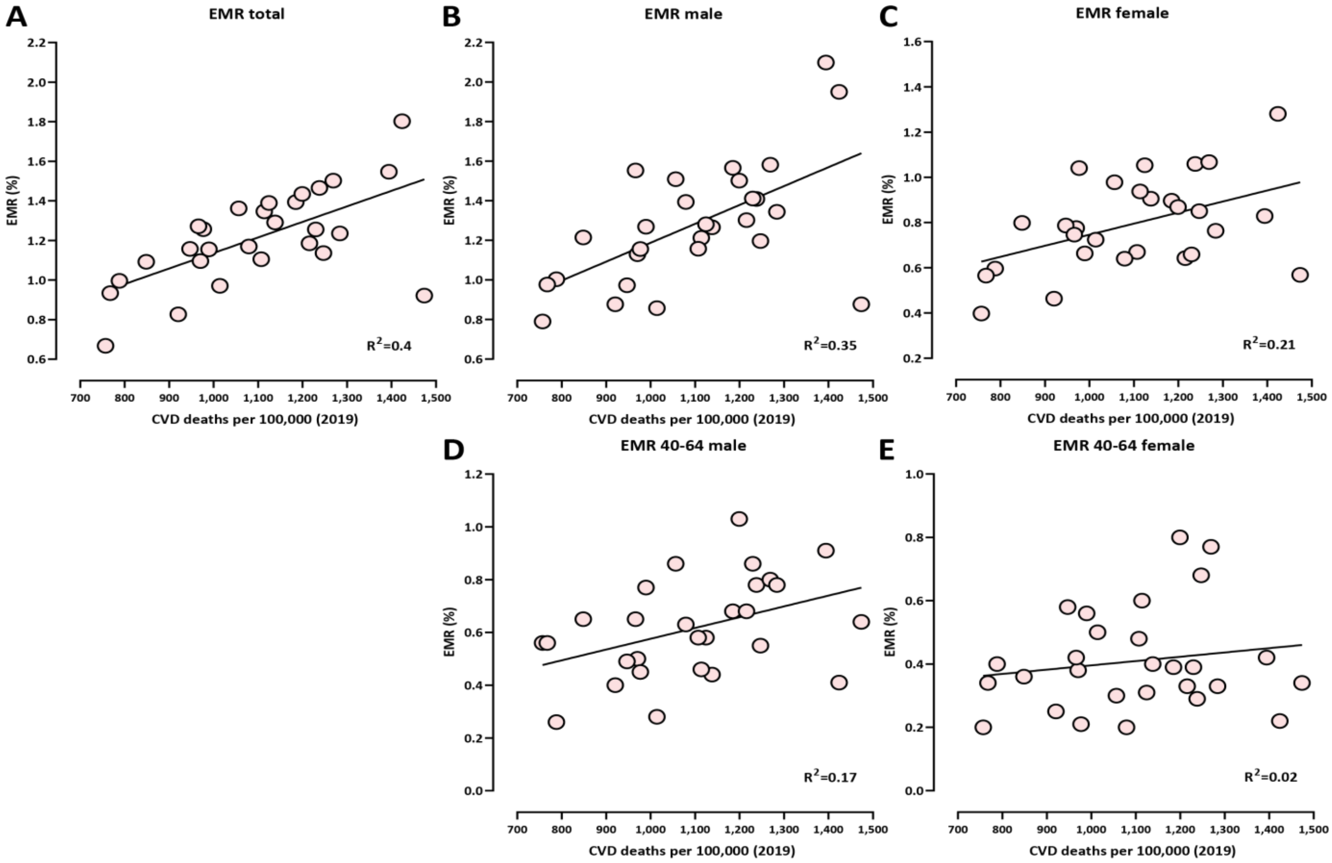

Supplementary Figure S6: Correlation between cardiovascular disease (CVD) prevalence (measured as the deaths from CVD per 100,000 people in 2019) and COVID-related excess mortality in Bulgarian regions (as measured by EMR). Shown is the Pearson R^2 correlation coefficient.

(A) total (Pearson $R^2 = 0.4$, $p = 0.0003$; Spearman $r = 0.59$, $p = 0.0008$);

(B) males (Pearson $R^2 = 0.35$, $p = 0.0009$; Spearman $r = 0.57$, $p = 0.0014$);

(C) females (Pearson $R^2 = 0.21$, $p = 0.0129$; Spearman $r = 0.43$, $p = 0.0196$);

(D) males ages 40-64 (Pearson $R^2 = 0.17$, $p = 0.026$; Spearman $r = 0.43$, $p = 0.0214$);

(E) females ages 40-64 (Pearson $R^2 = 0.02$, $p = 0.41$; Spearman $r = 0.12$, $p = 0.51$).

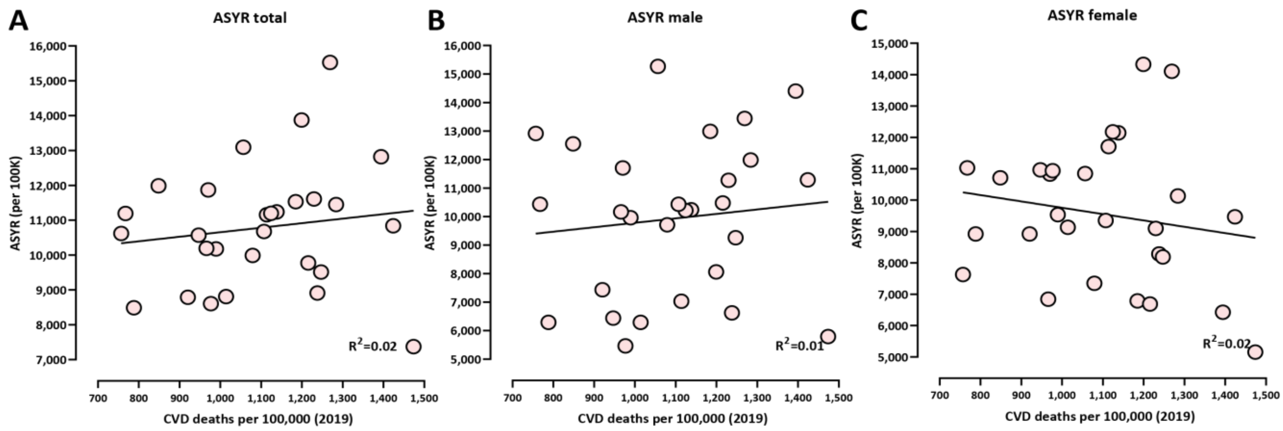

Supplementary Figure S7: Correlation between cardiovascular disease (CVD) prevalence (measured as the deaths from CVD per 100,000 people in 2019) and COVID-related excess mortality in Bulgarian regions (as measured by ASYR). Shown is the Pearson R^2 correlation coefficient.

(A) total (Pearson $R^2 = 0.02$, $p = 0.47$; Spearman $r = 0.18$, $p = 0.35$);

(B) males (Pearson $R^2 = 0.01$, $p = 0.31$; Spearman $r = 0.15$, $p = 0.44$);

(C) females (Pearson $R^2 = 0.03$, $p = 0.79$; Spearman $r = -0.16$, $p = 0.40$).
